# Synthesis, Antioxidant, Molecular Docking and DNA Interaction Studies of Metal-Based Imine Derivatives

**DOI:** 10.3390/molecules28155926

**Published:** 2023-08-07

**Authors:** Mohammad Ibrahim, Hazrat Un Nabi, Niaz Muhammad, Muhammad Ikram, Momin Khan, Musadiq Ibrahim, Abdullah F. AlAsmari, Metab Alharbi, Abdulrahman Alshammari

**Affiliations:** 1 Department of Chemistry, Abdul Wali Khan University Mardan (AWKUM) KPK, Mardan 23200, Pakistan; hazratunnabi@awkum.edu.pk (H.U.N.); drniaz@awkum.edu.pk (N.M.); ikram@awkum.edu.pk (M.I.); mominkhan@awkum.edu.pk (M.K.); 2 Department of Chemistry, Division of Biochemistry and Life Science, University of Glasgow, Glasgow G12 8QQ, UK; musadiqibrahim@gmail.com; 3 Department of Chemistry, Kohat University of Science and Technology, Kohat 26000, Pakistan; 4 Department of Pharmacology and Toxicology, College of Pharmacy, King Saud University, Riyadh 11451, Saudi Arabia; afalasmari@ksu.edu.sa (A.F.A.); mesalharbi@ksu.edu.sa (M.A.); abdalshammari@ksu.edu.sa (A.A.)

**Keywords:** Schiff base metal complexes, antioxidant, DNA binding, molecular docking

## Abstract

Currently, numerous ongoing studies are investigating the interaction of free radicals with biological systems, such as lipids, DNA and protein. In the present work, synthesis, characterization, antioxidant, DNA binding and molecular docking studies of Schiff base ligand and its Ni(II), Co(II), Cu(II) and Zn(II) were evaluated. The metal complexes have shown significant dose-dependent antioxidant activities higher than those of the free ligand but lesser than those of the standard antioxidant, ascorbic acid. The DNA binding constants (*K_b_*) were found in the order Zn(pimp)_2_ {9.118 × 10^5^ M^−1^} > H-pimp {3.487 × 10^5^ M^−1^} > Co(pimp)_2_ {3.090 × 10^5^ M^−1^} > Ni(pimp)_2_ {1.858 × 10^5^ M^−1^} > Cu(pimp)_2_ {1.367 × 10^5^ M^−1^}. Binding constants (*K_b_*) values calculated from the molecular docking analysis were found to be in close agreement with the experimental results. The obtained results indicate the importance of synthesis complexes as a source of synthetic antioxidants and anticancer drugs.

## 1. Introduction

Free radicals are generated within the body during normal metabolic activities and are markedly involved in many signaling pathways that control development and maintain cellular homeostasis [1,2]. However, the overproduction of these free radicals from both endo/exogenous sources has a great impact on humans in the etiology of various diseases [3]. The body possesses defense mechanisms against antioxidant nutrients and enzymes that arrest the damaging properties of the free radicals. However, continuous exposure to chemicals and contaminants may increase the amount of free radicals in the body beyond its ability to control and cause irreversible oxidative damage [4].

Therefore, antioxidants with free radical scavenging potential may be relevant in the therapeutic and prevention of diseases where free radicals are implicated. In addition to natural antioxidants such as vitamin C, vitamin E and carotenoid flavonoids, a number of cheaper and effective synthetic antioxidants have been prepared in research laboratories with promising antioxidant potential even higher than natural antioxidants in some cases [5].

An important class of compounds with excellent antioxidant potential appeared in the form of Schiff base transition metal complexes [6,7,8,9]. The azomethine (-HC=N) linkage of Schiff bases provides an opportunity for remarkable biological activities [10]. The lone pair of electrons on the nitrogen atom of the azomethine group has good chelating ability on Schiff bases, especially in combination with other donor atoms close to the azomethine group, and can result in the formation of stable transition metal complexes with simple synthesis and versatility and has a wide range of applications [7].

In addition to their antioxidant potential, Schiff base transition metal complexes have also shown encouraging binding abilities with DNA [6,8], a primary target for most chemotherapeutic agents. Bio-essential transition metals such as copper, cobalt and nickel complexes have shown their efficiencies as potential DNA binders and anticancer agents [9,10], thus offering a safe and more effective alternative to the available platinum-based drugs with higher toxicity, tumor resistance and severe side effects [10].

Keeping in view the importance of first-row transition metal Schiff base complexes, in the present study, synthesis, characterization, antioxidant, DNA binding activity and molecular docking studies of newly synthesized Schiff base ligand and its Co(II), Ni(II), Cu(II) and Zn(II) complexes were evaluated.

## 2. Results and Discussion

**Scheme 1:**
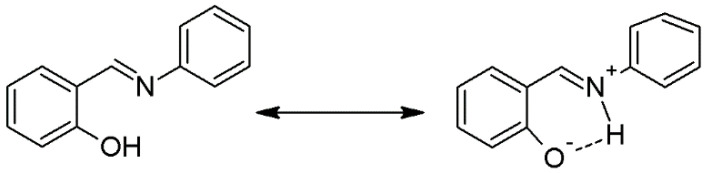
Intramolecular hydrogen bonding in H-pimp.

The Schiff base ligand 2-[(phenylimino)methyl]phenol (H-pimp) was synthesized by the condensation reaction of salicylaldehyde with aniline and characterized through different spectroscopic and analytical techniques. The infrared spectral data of the ligand (H-pimp) revealed a peak around 3200 cm^−1^ assigned to the NH stretch. The peak is weakly observed because of the possible intramolecular hydrogen bonding as shown in Scheme 1.

**Figure 1:**
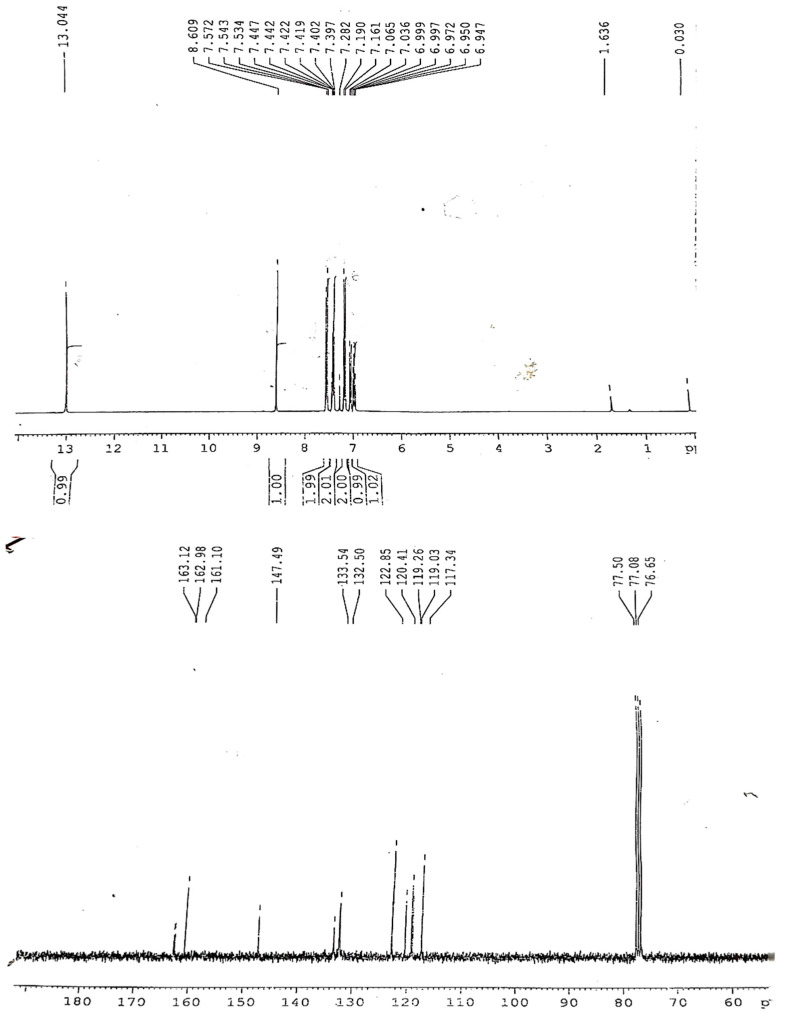
^1^H and ^13^C{^1^H}-NMR spectra of H-pimp.

The broad peak for hydroxyl stretch is obscured by the NH band appearance. The azomethine is observed at the expected position. The formation of the ligand is further supported by the NMR spectra. The ^1^H-NMR of the ligand (H-pimp) revealed a singlet signal assigned for each azomethine moiety and the phenolic hydroxyl group. The rest of the spectrum shows peaks for the aromatic protons. ^13^C-NMR shows an imine peak at 162 ppm, and the hydroxyl peak at 148 ppm (Figure 1). The Schiff base ligand reacted further with metal ions in a 2:1 molar ratio to produce metal derivatives of the anionic ligand with [M(pimp)_2_] compositions as shown in Scheme 1.

The IR spectra of the metal complexes share combine features of bonding irrespective of the metal center. The H-pimp ligand is behaving as a monoanionic ligand, offering coordination sites like O^-^ and HC=N. Both the stretching bands were seen displaced from the position observed in the spectrum of the free ligand. The rest of the spectra show differences due to the complexation. The elemental compositions in metal complexes also revealed closeness for the [M(pimp)_2_] compositions in all the metal complexes. Therefore, it has been observed unambiguously that metal complexation occurs through the bonding of two molecules of the ligand with metal centers. The metal complexes were also observed to be non-electrolyte in nature.

The UV-visible spectra of the metal complexes were recorded within the region 320–1000 nm in a methanolic solution. The Co(pimp)_2_ complex showed a single and broadband at 860 nm assigned to the distorted square planar ^2^A_2g_ →^2^B_1g_ transition. The other transitions like ^1^A_1g_→^1^B_1g_ and ^1^A_1g_→E_g_ were considered enfolded within the broad ^2^A_2g_ →^2^B_1g_ transition. Similarly, the visible spectrum of Ni(pimp)_2_ metal complex also revealed a single transition ^1^A_1g_ → ^1^A_2g_ for the distorted square planer geometry. The Cu(pimp)_2_ showed a weak transition at 660 nm assigned to the dz^2^ → dx^2^ − y^2^ in a distorted tetrahedral geometry. All the transitions in metal complexes were assigned charge transfer ligands to metal transitions where the imine group is involved in donating the electron density towards metal orbitals.

### 2.1. In Vitro Antioxidant Potential of the Synthesized Compounds

**Figure 2:**
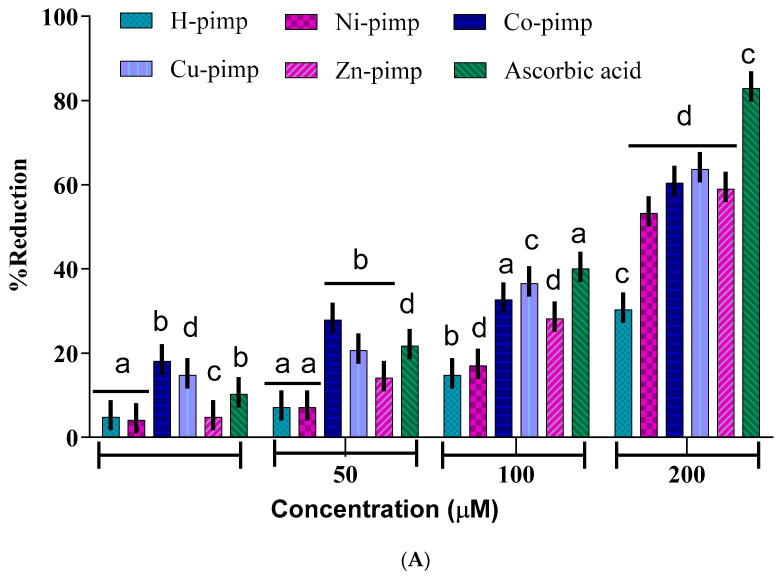

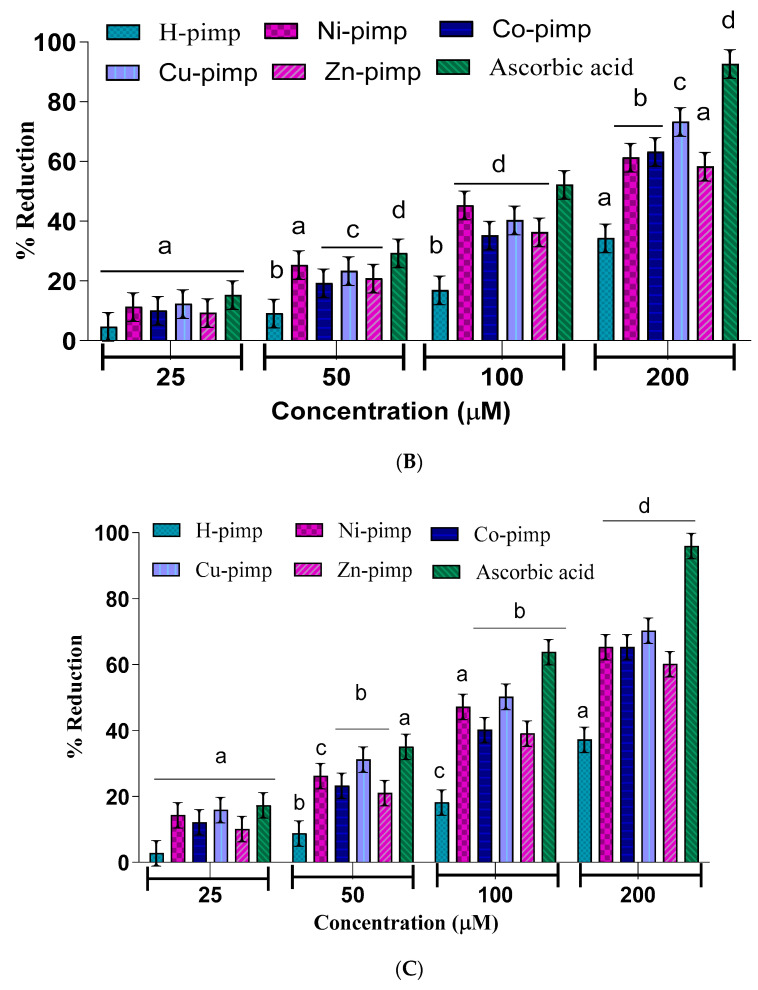

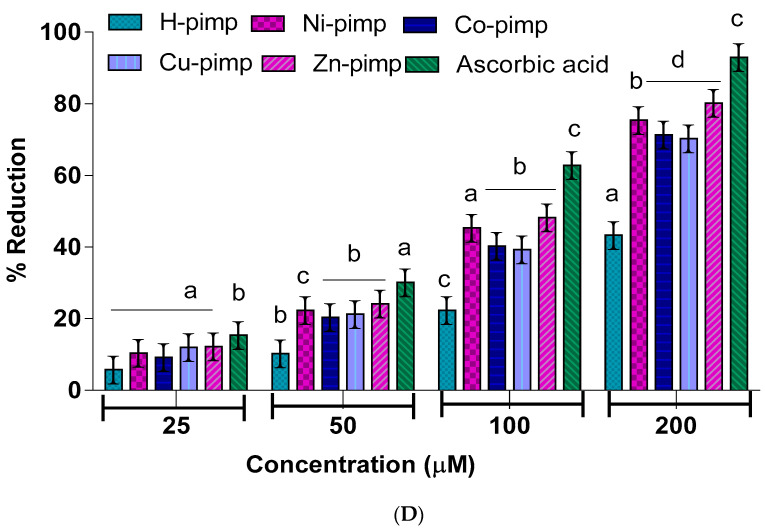
(**A**) DPPH radical scavenging activity of Ligand and their metal complexes. (Ligand, Ni(II) Complex, Co(II) Complex, Cu(II) Complex, Zn(II) Complex and Ascorbic acid). Mean ± SEM was used to express the values Significantly ^a^ *p* < 0.0006, ^b^ *p* < 0.0017, ^c^ *p* < 0.0027, ^d^ *p* < 0.0086. (**B**) Ferric ion reducing the activity of Ligands and their metal complexes. Values are expressed as mean ± SEM (triplicate tests were conducted for each sample). Mean ± SEM was used to express the values significantly ^a^ *p* < 0.0006, ^b^ *p* < 0.0017, ^c^ *p* < 0.0027, ^d^ *p* < 0.0086. (**C**) Phosphomolybdenum assay of Ligand and their metal complexes. Values are expressed as mean ± SEM (triplicate tests were conducted for each sample). Mean ± SEM was used to express the values Significantly ^a^ *p* < 0.0006, ^b^ *p* < 0.0017, ^c^ *p* < 0.0027, ^d^ *p* < 0.0086. (**D**) Hydroxyl scavenging assay of Ligands and their metal complexes. Values are expressed as mean ± SEM (triplicate tests were conducted for each sample). Mean ± SEM was used to express the values Significantly ^a^ *p* < 0.0006, ^b^ *p* < 0.0017, ^c^ *p* < 0.0027, ^d^ *p* < 0.0086.

The antioxidant potential of the Schiff base ligand H-pimp and its synthesized metal complexes M(pimp)_2_ was checked by different assays, including a DPPH-free radical scavenging assay, a ferric ion reducing power assay (FRAP assay), total antioxidant activity (TAA assay) and hydroxyl radical scavenging activity (•OH assay) against ascorbic acid as a standard according to spectrophotometric measurements. The experimental findings presented in Figure 2A–D have shown dose-dependent antioxidant potential for the synthesized compounds. The antioxidant potential data in terms of IC_50_ values for the studied assays are given in Table 1.

The Figure 2A–D shows the concentration of the reducing agent required to produce 50% scavenging activity. A lower IC_50_ value corresponds to a higher free radical scavenging or antioxidant activity.

The IC_50_ value data shown in Table 1 reflect the ligand H-pimp and its transition metal complexes M(pimp)_2_, where M = Cu^2+^, Ni^2+^, Co^2+^ and Zn^2+^ show lesser antioxidant potential compared to the standard ascorbic acid in all assays. Another important point is the higher antioxidant potential of the synthesized metal complexes compared to the free ligand H-pimp. Figure 2A–D shows that the higher antioxidant potential after complexation could be attributed to the presence of an electropositive metal center, resulting in the activation of π electronic cloud of the benzene ring of the ligand center for antioxidant activity. In addition, the chelate formation during complexation results in the redistribution of the electronic cloud over the whole chelate ring, which could enhance the electron-donating ability of the complex, hence its antioxidant potential. Among metal complexes, the highest antioxidant potential was shown by the copper complex Cu(pimp)_2_ in all assays in Figure 2A–D. The zinc complex Zn(pimp)_2_ was found to be relatively lower in antioxidant potential, probably due to the redox inert closed stable 3d^10^ electronic configuration for the Zn^2+^ ion. The synthesized metal complexes have the same metal-to-ligand ratio of (1:2), so the difference in the activity could be attributed to the differences in their electronic configuration. 

### 2.2. DNA Binding Activity

Metal-based complexes have offered an efficient solution for the treatment of cancer, a lethal disease caused by rapid DNA replication. Mostly, metal complexes directly interact with DNA, alter its shape and thus stop its rapid replication and, hence, cancer. This makes the study of complex-DNA interaction an important research area to overcome the life-threatening disease, cancer. 

Electronic absorption spectroscopy can be used as a preliminary technique to study the metal complex-DNA interaction. Metal complex-DNA interaction alters UV-Vis spectra of the complexes in terms of λ_max_ shift (blue/red) or intensity changes (hypo/hyperchrpmisim). The extent of wavelength shift or change in absorption intensity shows the strength of interaction. The hypochromisim is associated with a red shift observed in the complex spectra. The increasing concentration of DNA during electronic absorption spectroscopic titrations indicates complex-DNA interaction in the form of intercalation [11,12]. This interaction is a consequence of stacking interactions between an aromatic chromophore of the complex and the DNA base pairs. After intercalation, the π* orbital of the complexes ligand couple with π orbitals of the base pairs of DNA. This coupling results in a decrease in energy required for π-π* transition and causes a red shift. If the coupling π* orbitals are partially filled by the electrons then transition probabilities also decrease causing hypochromisim [10].

**Figure 3:**
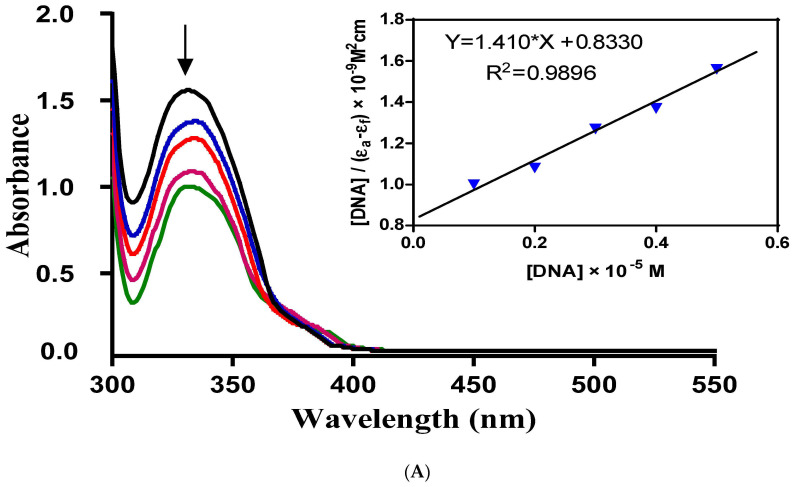

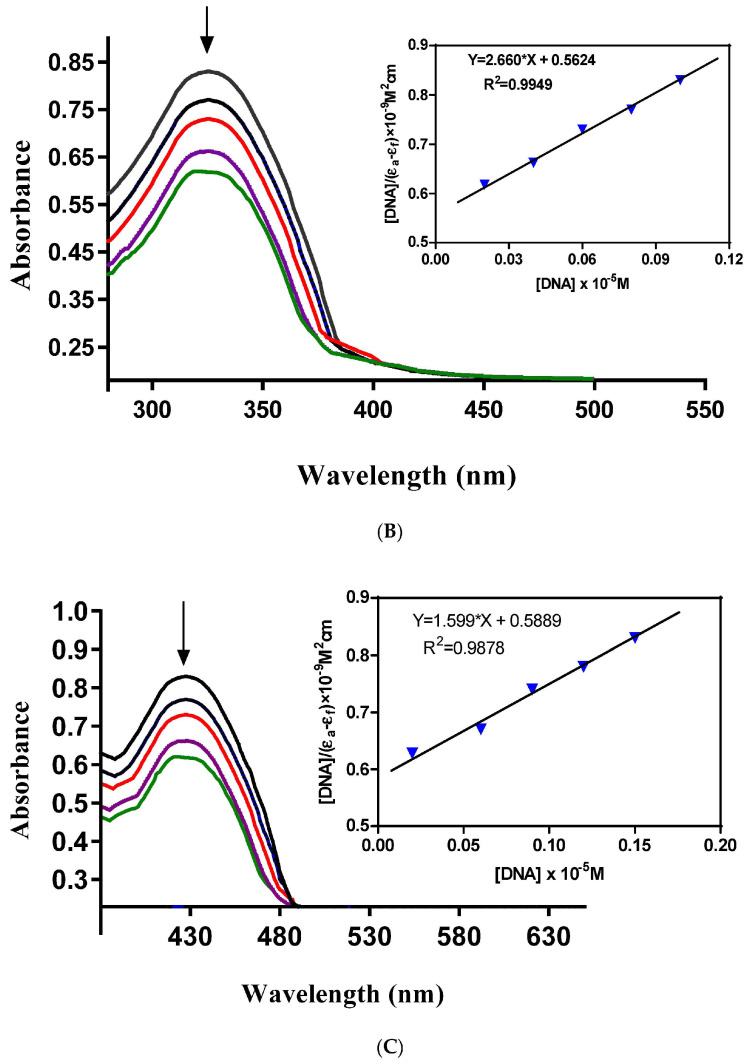

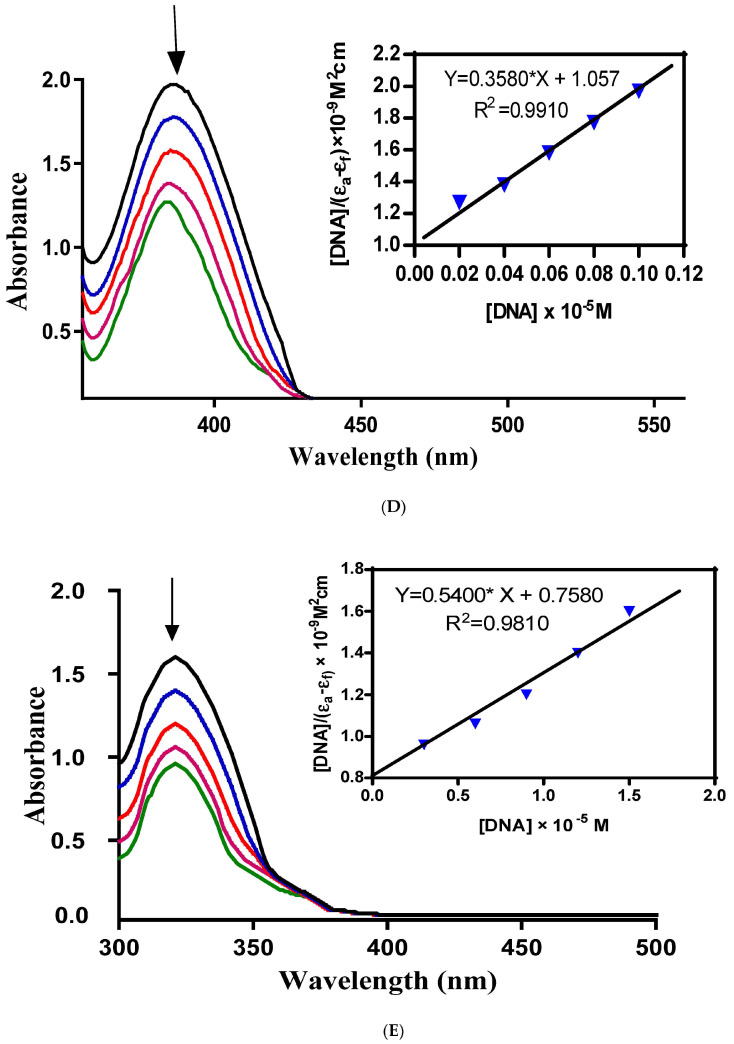
(**A**). Absorption spectra of H-pimp in buffer pH 7.2 at 25 °C in the presence of increasing amount of DNA. Arrows indicate the changes in absorbance upon increasing the DNA concentration. Inset: plot of [DNA]/(Ɛa − Ɛ_f_) × 10^−9^ M^2^cm versus [DNA] × 10^−5^ M for titration of DNA with H-pimp. (**B**) Absorption spectra of Ni-pimp in buffer pH 7.2 at 25 °C in the presence of increasing amount of DNA. Arrows indicate the changes in absorbance upon increasing the DNA concentration. Inset: plot of [DNA]/(Ɛa − Ɛ_f_) × 10^−9^ M^2^cm versus [DNA] × 10^−5^ M for titration of DNA with Ni-pimp. (**C**) Absorption spectra of Co-pimp in buffer pH 7.2 at 25 °C in the presence of increasing amount of DNA. Arrows indicate the changes in absorbance upon increasing the DNA concentration. Inset: plot of [DNA]/(Ɛa − Ɛ_f_) × 10^−9^ M^2^cm versus [DNA] × 10^−5^ M for titration of DNA with Co-pimp. (**D**) Absorption spectra of Cu-pimp in buffer pH 7.2 at 25 °C in the presence of increasing amount of DNA. Arrows indicate the changes in absorbance upon increasing the DNA concentration. Inset: plot of [DNA]/(Ɛa − Ɛ_f_) × 10^−9^ M^2^cm versus [DNA] × 10^−5^ M for titration of DNA with Cu-pimp. (**E**) Absorption spectra of Zn-pimp in buffer pH 7.2 at 25 °C in the presence of increasing amount of DNA. Arrows indicate the changes in absorbance upon increasing the DNA concentration. Inset: plot of [DNA]/(Ɛa − Ɛ_f_) × 10^−9^ M^2^cm versus [DNA] × 10^−5^ M for titration of DNA with Zn-pimp.

In order to assess the DNA-binding potential of the synthesized Schiff base ligand (H-pimp) and its Cu(pimp)_2_, Ni(pimp)_2_, Co(pimp)_2_ and Zn(pimp)_2_ complexes, electronic absorption spectral titrations were performed with fixed and variable concentrations of synthesized compounds and DNA, respectively. The results of the spectroscopic titrations are shown in Figure 3A–E and for H-pimp, Ni(pimp)_2_, Co(pimp)_2_, Cu(pimp)_2_ and Zn(pimp)_2_, respectively, indicating hypochromism associated with a very small red shift with increasing concentrations of DNA solutions, thus indicating intercalation [13]. 

In order to find the extent of this intercalative interaction between synthesized compounds and DNA, the intrinsic binding constants (*K_b_*) values were calculated according to the equation {[DNA]/(Ɛ_a_ − Ɛ_f_) = DNA/(Ɛ_a_ − Ɛ_f_) + 1/[*K_b_*(Ɛ_b_ − Ɛ_f_)]} [10]. In the equation, Ɛ_a_, Ɛ_f_, and Ɛ_b_ are the apparent, free and bound compound extinction coefficients, respectively. A plot of [DNA]/(Ɛ_b_ − Ɛ_f_) versus [DNA], gave a slope of 1/(Ɛ_b_ − Ɛ_f_) and a Y-intercept equal to [*K_b_*/(Ɛ − Ɛ_f_)]^−1^; *K_b_* is the ratio of the slope to the Y-intercept.

The intrinsic binding constants showing the binding strength of the compounds with DNA varied in the order: Zn(pimp)_2_ {9.118 × 10^5^ M^−1^}> H-pimp {3.487 × 10^5^ M^−1^} > Co(pimp)_2_ {3.090 × 10^5^ M^−1^} > Ni(pimp)_2_ {1.858 × 10^5^ M^−1^} > Cu(pimp)_2_ {1.367 × 10^5^ M^−1^}. The binding strength order seems to be related to the electronic configuration of the complexes. Zinc(pimp)_2_ with a closed 3d^10^ symmetric configuration shows the highest binding strength with DNA. The calculated *K_b_* values were found slightly higher than a classical intercalator, cisplatin (5.71 × 10^4^ M^−1^) [9].

### 2.3. Structural Analysis and Molecular Docking 

**Figure 4:**
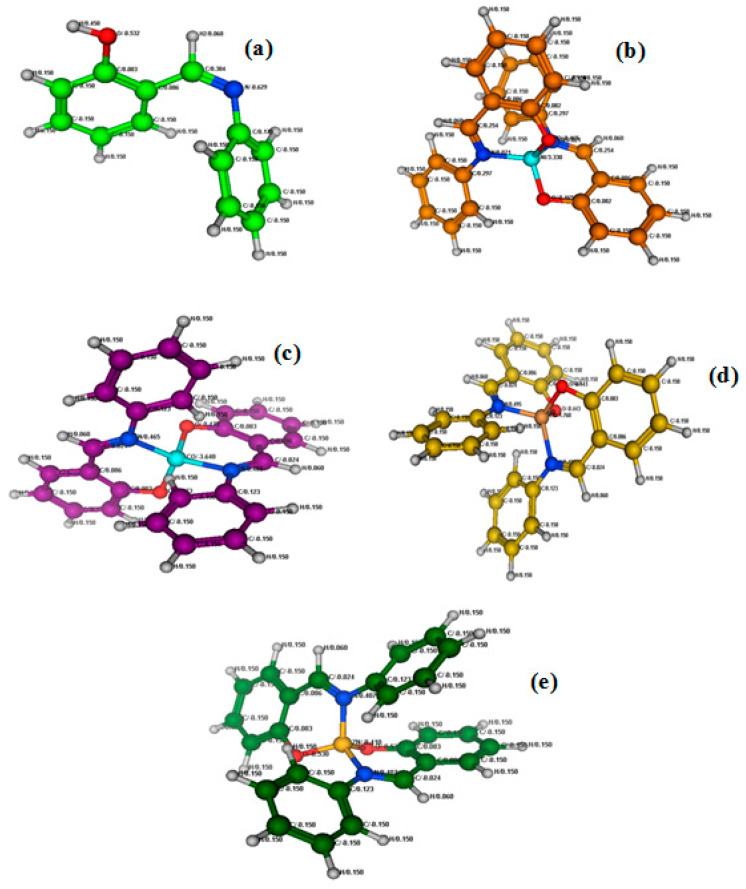
Optimized molecular structures of Schiff base (Z)-2-((phenylimino) methyl)phenol Ligand (**a**) and their metal (**b**) Ni(II), (**c**) Co(II), (**d**) Cu(II) and (**e**) Zn(II)) complexes.

**Figure 5:**
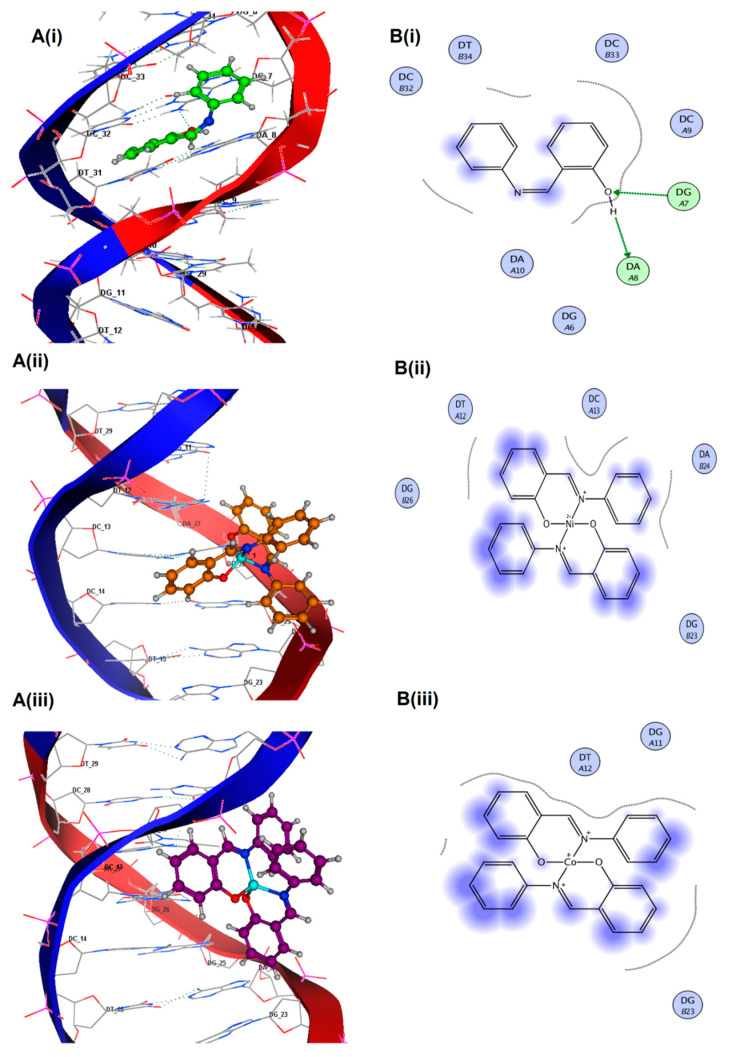

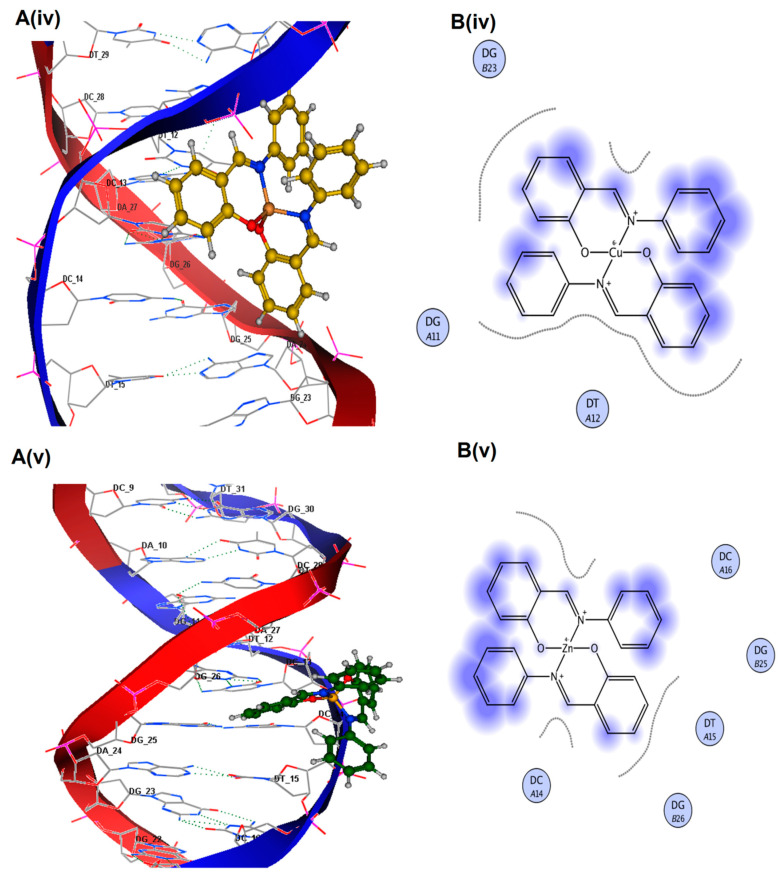
Molecular docked complex of 1D66 with Schiff base (Z)-2-((phenylimino) methyl) phenol Ligand and their metal complexes with Ni(II), Co(II), Cu(II) and Zn(II) {**A**(**i**–**v**)}. 2D ligplot showing interactions of compounds with base pairs of 1D66 {**B**(**i**–**v**)}.

The structural analysis of 2-{(phenylimino) methyl}phenol ligandH-pimp and its metal complexes M(pimp)_2_ was carried out using a semiempirical PM3 method (Figure 4a–e) for their charge distribution and molecular docking with doubly stranded DNA (PDB:1D66). The molecular docking studies determined all possible configurations of computationally anticipated Schiff base ligand H-pimp, Ni(pimp)_2_, Co(pimp)_2_, Cu(pimp)_2_, and Zn(pimp)_2_ complexes with DNA to understand the molecular mechanism and physical mode of interaction. DNA is the primary pharmacological target of a number of anticancer compounds. Thus, the interaction between DNA and metal complexes is of vital prominence in understanding the mechanism. Transition metal complexes interact with DNA via both covalent and/or non-covalent interactions. Non-covalent DNA interactions include intercalative, electrostatic and groove binding of metal complexes with a DNA helix [11]. A pose view analysis of H-pimp and its metal complexes was also performed to substantiate the mode of DNA binding, shown in Figure 4a–e. Least energy confirmation pose of H-pimp revealed that the planar aromatic part of the ligand intercalates between stacked base pairs of DNA by π-stacking interactions. The 2D lig plot of the ligand showed that H of the H-O group develops H-bonding with adenine DA (A8) of DNA, whereas O of the H-O group builds H-bonding with H atom of Guanine DG(A7), as shown in Figure 5A(i–v),B(i–v). H-bonding characteristics of ligands rendered significantly high binding affinity with DNA, as shown in Table 2. Ni(II) complex of ligand exhibited mixed mode of intercalation and groove binding. It can be seen that the Ph-ring of the Zn(II) complex intercalates between DNA base pairs while the rest of the bulky structure latches onto the minor groove of DNA, establishing Vander Waal’s interactions with grooves, as visible in Figure 5B(ii). It was observed that Co(II) and Cu(II) complexes of H-pimp fit well into the minor groove of DNA via hydrophobic and Vander Waal’s interactions, as shown in Figure 5B(iii,iv). The interactions of the Zn(II) complex with DNA are attributed to the intercalation of the aromatic planar ring with flanking base pairs and, more importantly, with DC(A14), DG(B26) and DT(A15) of DNA, as can be seen in Figure 5B(v). A substantial observation was that the Zn(II) complex showed enhanced interactions and binding affinity in terms of the highest *K_b_* value as compared to free ligands and other complexes, as shown in Table 2. The negative values of free energy depicted that the physical interaction mechanism of compounds with DNA has been proven to be spontaneous.

For the comprehension of microscopic interactions between DNA and H-pimp ligand as well as its complexes, a number of physicochemical descriptors were calculated, listed as electronic descriptors and steric descriptors as shown in Table 3 and Table 4, respectively.

Electronic parameters of the ligand and complexes were calculated; however, semi-empirical calculations of transition metal compounds were complicated due to partially filled d-orbitals of the metal ions that are responsible for the multifarious structures with a large variety of possible coordination numbers and geometries [14,15]. Hence, electronic parameters of only ligand and Zn complex (completely filled d-orbital) were determined. E_HOMO_ and E_LUMO_ values gave an estimate of the electron-donating or electron-accepting character of a given compound. Consequently, a compound is considered more electron-donating as its E_HOMO_ value increases and more electron-accepting as its E_LUMO_ value decreases. Table 3 depicts that Zn(II) complex is more electron donating as compared to due to the higher value of its E_HOMO_.

**Figure 6:**
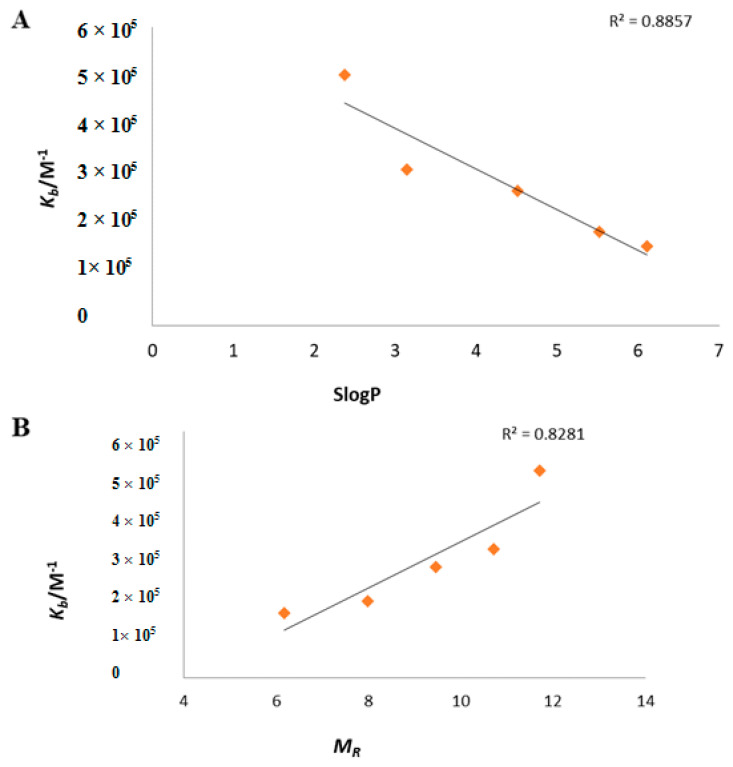
(**A**) Plot of formation constant *K_b_* vs. Partition Coefficient (SlogP). (**B**) Plot of formation constant *K_b_* vs. Molar Refractivity (*M_R_*).

An excellent correlation of two steric parameters, i.e. partition coefficient (log *p*) and Molar refractivity (M_R_) with binding constant (*K_b_*), has been perceived. The partition coefficient (log *p*) is illustrative of the hydrophobicity of the molecule. In this work, *slogP* revealed a reasonable inverse correlation with the *K_b_* of ligand and its metal complexes (R^2^ = 0.885), indicating that the compounds with a lower *slogP* are anticipated to constitute stronger complexes, as shown in Figure 6A.

Another imperative steric descriptor calculated in the present work was Molar refractivity (*M_R_*), a measure of the total polarizability of a mole of a substance and its dependence on the temperature, the index of refraction and the pressure. A direct correlation of the *M_R_* (R^2^ = 0.8281) with *K_b_* was indicative of the fact that compounds with higher *M_R_* have more binding affinity with DNA (Figure 6B).

## 3. Materials and Methods

### 3.1. Chemicals and Reagents

Analytical grade salicylaldehyde, aniline, 2,2-diphenyl-1-picrylhydrazyl radical (DPPH), ascorbic acid, tris-HCl buffer, ferric chloride, O-phenenthroline, sulfuric acid, ammonium molybdate, potassium phosphate (monophosphate and di-phosphate), hydrogen peroxide, ethanol and salmon sperm DNA (SSDNA) were purchased from commercial suppliers and were used without any further purification.

### 3.2. Synthesis of 2-[(Phenylimino)methyl]phenol{H-pimp}

The Schiff base ligand (H-pimp) was synthesized by reacting equimolar (5 mmol) methanolic solutions of aniline and salicyladehyde as shown in Scheme 1. A yellowish product was produced instantaneously upon mixing which was filtered and recrystallized from a concentrated metahanolic solution. 

Yield: 63%, M.pt: 48 °C, Elemental analysis (C_13_H_11_NO) Calc. (Exp.): C, 79.16 (80.63); H, 5.62 (5.12); N, 7.10 (7.01). IR (cm^−1^): 3200 (w), 1569 (s), 1483 (s), 1407 (s), 1363 (s), 1282 (s), 1156 (s), 1110 (s), 987 (s), 912 (s), 857 (s), 756 (s), 688 (s), ^1^H-NMR (300.13 MHz, CDCl_3_, 303K): δ = 6.94 (d, ^3^*J*_HH_ = 7.03 Hz, 1H, H13), 6.97 (d, ^3^*J*_HH_ = 7.03 Hz, 2H, H12 & H14), 6.99 (d, ^3^*J*_HH_ = 7.01 Hz, 2H, H4 & H5), 7.02 (d, ^3^*J*_HH_ = 7.2 Hz, 1H, H3), 7.16 (d, ^3^*J*_HH_ = 7.31 Hz, 2H, H11 & H15), 7.28 (d, ^3^*J*_HH_ = 6.9 Hz, 1H, H6), 8.60 (s, Ar HC=N), 13.0 (s, OH), ^13^C{^1^H}-NMR (75.47 MHz, CDCl_3_, 303k), 117 (CH, C13), 119 (CH, C12 & C14), 119.2 (CH, C4 & C5), 120 (CH, C3), 122 (CH, C11 & C15), 132 (CH, C6), 133 (C, C2), 148 (C, C1), 161 (C, C10), 163 (CH, Ar HC-N).

### 3.3. Synthesis of Metal Complexes {M(pimp)_2_}

**Scheme 2:**
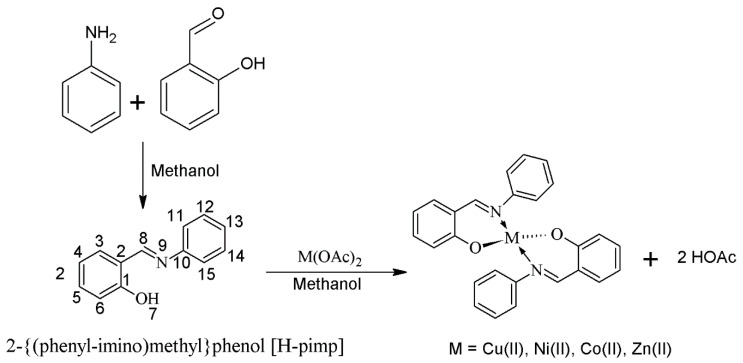
Metal complexation of H-pimp Schiff base ligand.

The transition metal complexes of the Schiff base ligand (H-pimp) were synthesized (Scheme 2) by a reported procedure [11]. The metal salts M(OAc)_2_ were initially dehydrated by keeping them in an oven for 3–4 h at 110 °C. A methanolic solution of ligand H-pimp (5 mmol) was added to the methanolic solution of metal acetate (2.5 mmol) and stirred for 3 h. The product was obtained either instantly or upon concentrating the solution through a rotary evaporator.

### 3.4. Bis-(2-[(phenylimino)methyl]phenolate)cobalt(II) {(Co-pimp)_2_}

Yield: 43%. Elemental analysis (C_26_H_20_CoN_2_O_2_) Calc. (Exp.): C, 69.18 (69.88); H, 4.47 (5.11); Co, 13.06 (13.02); N, 6.21 (6.12)%. IR (cm^−1^): 1536 (s), 1482 (s), 1465 (s), 1450 (s), 1328 (s), 1180 (s), 1148 (s), 1122 (s), 1086 (s), 1010 (s), 976 (s), 929 (s), 858 (s), 837 (s), 758 (s), 698 (s), λ_max_ = 860 nm (ε = 17.6 M^−1^ cm^−1^, ^2^A_2g_ → ^2^B_1g_).

### 3.5. Bis-(2-[(phenylimino)methyl]phenolate)nickel(II) {(Ni-pimp)_2_} 

Yield: 55%. Elemental analysis (C_26_H_20_N_2_NiO_2_) Calc. (Exp.): C, 69.22 (69.28%); H, 4.47 (4.90%); N, 6.21 (6.29%); Ni, 13.01 (12.11%). IR (cm^−1^): 1533 (s), 1464 (s), 1443 (s), 1416 (s), 1343 (s), 1260 (s), 1224 (s), 1181 (s), 1147 (s), 1123 (s), 1033 (w), 981(s), 946 (s), 871 (w), 821 (s), 810 (s), 761 (s), 751 (s), 728 (s), 689 (s), 671 (s), λ_max_ = 690 nm (ε = 28.3 M^−1^ cm^−1^, ^1^A_1g_ → ^1^A_2g_).

### 3.6. Bis-(2-[(phenylimino)methyl]phenolate)copper(II) {(Cu-pimp)_2_} 

Yield: 68%. Elemental analysis (C_26_H_20_CuN_2_O_2_) Calc. (Exp.): C, 68.48 (69.33%); H, 4.42 (4.92%); Cu, 13.94 (13.01%); N, 6.14 (7.44%)%. IR (cm^−1^): 1555 (s), 1523 (s), 1478 (s), 1441 (s), 1388 (s), 1351 (s), 1323 (s), 1255 (s), 1205 (w), 1175 (s), 1151 (s), 1133 (s), 1098 (s), 1031 (s), 1009 (s), 987 (s), 937 (s), 834 (s), 767 (s), 699 (s), 623 (s), λ_max_ = 660 nm (ε = 188.6 M^−1^ cm^−1^, dz^2^ → dx^2^ − y^2^ ).

### 3.7. Bis-(2-[(phenylimino)methyl]phenolate)zinc(II) {(Zn-pimp)_2_}

Yield: 55%. Elemental analysis (C_26_H_20_N_2_O_2_Zn) Calc. (Exp.): C, 68.20 (70.01%); H, 4.40 (4.78%); N 6.12 (6.89%); Zn, 14.29 (14.95%). IR (cm^−1^): 1581 (s), 1531 (s), 1459 (s), 1441 (s), 1389 (s), 1351 (s), 1326 (s), 1253 (s), 1169 (s), 1151 (s), 1096 (s), 1032 (s), 1008 (s), 927 (s), 829 (s), 789 (s), 762 (s), 688 (s), 596 (s).

### 3.8. In Vitro Antioxidant Studies

The synthesized compounds were studied for their in vtiro antioxidant potential by different assays using UV-Visible Spectrophotometer. In DPPH radical scavenging assay, different concentrations (25, 50, 100 and 200 µM) of compounds were mixed with an ethanolic solution containing 85 µM DPPH radical. The decrease in absorbance was measured at 517 nm. Ascorbic acid was used as a positive control to determine the maximal decrease in DPPH absorbance. The values are expressed in the percentage of inhibition of DPPH absorbance in relation to the control values without the compounds (ascorbic acid maximal inhibition was considered 100% of inhibition).

The ferric ion-reducing power of the newly synthesized compounds was determined according to the reported procedure [12,13]. Varying concentrations (25, 50, 100 and 200 µM) of compounds, 0.2 mL of 3.6 mM ferric chloride, 0.3 mL of 100 mM tris buffer (pH = 7.4), 0.1 mL of 9 mM O-phenanthroline were mixed and diluted up to 3.0 mL with ultra-pure distal water. Solutions were shaken for 10 min vigorously and left to stand at room temperature. The increase in absorbance of the sample solution was measured at 595 nm. Ascorbic acid at the same concentrations was utilized as a reference standard and without a compound sample mixture as a control. 

The total antioxidant capacity of the compounds was evaluated using a phosphomolybdenum assay [10] based on the conversion of Mo(VI) into Mo(V), with the test compound acting as a reducing agent in comparison with ascorbic acid used as a standard.

The reagent solution containing varying concentrations (25, 50, 100 and 200 µM) of synthesized compounds aliquot in ethanol, 0.7 mL of 0.6 M sulphuric acid, 1.0 mM ammonium molybdate, 1.0 mL of 28 Mm pottasium pasphate and ultra-pure distal water were incubated at 95 °C for 90 min. After cooling, at room temperature, the increase in absorbance of the mixture was measured at 695 nm. Ascorbic acid was utilized as the reference standard and without compounds sample mixture as control. 

The scavenging activity of the synthesized compounds for hydroxyl radicals was measured with the Fenton reaction [9]. Varying concentrations (25, 50, 100 and 200 µM) of synthesized compounds, 0.1 mL of 7.5 mM O-phenanthroline, 0.5 mL of 0.2 M phosphate buffer ( pH 6.6), 0.1 mL of 7.5 mM ferrous sulfate and 0.1 mL of H_2_O_2_ (0.1%) were mixed and diluted up to 3 mL with distilled water. The reaction mixture was incubated at room temperature for 30 min and the absorbance was measured at 510 nm. The reaction mixtures without synthesized compounds have been used as control and without compounds and H_2_O_2_ as a blank. 

### 3.9. DNA Absorption Spectroscopic Studies

The electronic absorption spectroscopy was used to study the interaction between synthesized compounds and salmon sperm DNA (SSDNA). The solution of SSDNA in the buffer 50 mM NaCl/5 mM Tris–HCl (pH 7.2) in water gave an absorbance ratio of 1.9 at 260/280 nm, indicating that the DNA was sufficiently free from protein [11]. The concentration of DNA was measured using its extinction coefficient at 260 nm (6600 M^−1^ cm^−1^) after 1:100 dilution. Concentrated stock solutions of the synthesized compounds were prepared by dissolving the compounds in ethanol and diluting them suitably with the corresponding buffer to the required concentration for all of the experiments. 

### 3.10. Statistical Analysis

Linear regression analysis was used to calculate IC_50_ ± SEM values from data and graphs by using Graph pad prism 6^®^. Significant differences among the means of data were tested by the one-way ANOVA followed by the student’s *t*-test with significance level (*p* < 0.05). All the tests were conducted in triplicate.

### 3.11. Molecular Docking Methodology

The chemical structures of the Schiff base ligand and its complexes with Co(II), Ni(II), Cu(II) and Zn(II) were sketched and optimized on the MOE2017 window using MOE molecular builder and were entered into the MOE database. The 3-D structure of DNA with PDB ID-1D66, resolution of 2.7, and sequence of CCGGAGGACTGTCCTCCGG was obtained from the RCSB protein Data Bank [16]. For the purpose of docking simulation, PDB internal coordinates of DNA were optimized using a molecular dynamic AMBER force field and semi-empirical PM3 approaches to attain minimum energy and stable conformation. Water molecules were removed from DNA structures using a sequence editor of MOE to exclude the effect of water on the interaction of DNA with the ligand and its metal complexes. Structures were protonated with their standard geometry followed by their energy optimization tool using MOPAC 7.0. The resulting structures were subjected to a systematic conformational search at default parameters with an RMS gradient of 0.01 kcal/mol using Site Finder to find out active sites of DNA molecules. Finally, a few docking runs were carried out to get a final binding pose from the scoring function. The best conformation was selected based on energetic ground and the minimum Final Docking Energy (∆G) [17].

## 4. Conclusions

In the present work, preliminary screening of a Schiff base ligand and its transition metal complexes has shown interesting antioxidant and DNA binding activities. Doze-dependent antioxidant potential was shown by the compounds. IC_50_ values for the studied antioxidant assays were significantly reduced after complexation, reflecting the role of the metal center in the antioxidant activity. UV-vis spectroscopic results for the DNA interaction of the synthesized compounds showed hypochromism associated with a very small red shift, indicating intercalation of binding strength in the order Zn(pimp)_2_ {9.118 × 10^5^ M^−1^} > H-pimp {3.487 × 10^5^ M^−1^} > Co(pimp)_2_ {3.090 × 10^5^ M^−1^} > Ni(pimp)_2_ {1.858 × 10^5^ M^−1^}> Cu(pimp)_2_ {1.367 × 10^5^ M^−1^}. Among complexes, metal electronic configuration seems to be the controlling factor for the antioxidant activity and DNA binding strength. Molecular docking studies revealed the DNA-compound interactions due to the ability of the synthesized compounds to develop H-bonding, hydrophobic and Vander Waal’s interactions. With a decrease in hydrophobicity and an increase in total polarizability of the synthesized compounds, binding affinity with DNA was increased. 

## Figures and Tables

**Table 1:** The IC_50_(µM) ± SEM values of Ligand (H-pimp) and its metal complexes M(pimp)_2_ ^a^ for different assays.

Compound	DPPH Assay	FRAP Assay	TAA Assay	OH Assay
H-pimp	333.58 ± 5.771	293.28 ± 6.538	264.41 ± 7.532	49.63 ± 8.391
Ni(pimp)_2_	196.70 ± 11.30	145.90 ± 11.00	134.78 ± 11.29	39.41 ± 14.32
Co(pimp)_2_	159.44 ± 9.066	153.66 ± 11.65	134.42 ± 11.55	43.19 ± 13.62
Cu(pimp)_2_	150.51 ± 10.95	130.47 ± 13.31	120.93 ± 11.76	36.04 ± 12.84
Zn(pimp)_2_	170.34 ± 11.85	162.90 ± 10.61	155.77 ± 11.76	36.91 ± 15.01
Ascorbic acid(AA)	121.07 ± 15.94	99.612 ± 16.91	86.729 ± 17.21	29.25 ± 17.21

^a^ Standard used is Ascorbic acid (AA).

**Table 2:** Free energy (∆G), binding constant (K_b_) of ligand and its metal complexes calculated from molecular docking data.

Complex	Free Energy (−∆G)/kJ/mol	Binding Constants (*K_b_*)/M^−1^
H-pimp	31.56	3.14 × 10^5^
Ni(pimp)_2_	30.29	1.88 × 10^5^
Co(pimp)_2_	31.22	2.73 × 10^5^
Cu(pimp)_2_	29.87	1.59 × 10^5^
Zn(pimp)_2_	32.79	5.04 × 10^5^

**Table 3:** Data set of selected electronic descriptors calculated from molecular docking data.

Complex	E_Total_/kcalmol	E_HOMO_/kcal/mol	E_LUMO_/kcal/mol	E_electro_/kcal/mol	E_vandr_/kcal/mol	E_IP_/kcal/mol
H-pimp	−449,908.307	−8.8801	3.9700	−296,836.46	0.00000	8.8801
Ni(pimp)_2_	-	-	-	-	0.00000	-
Co(pimp)_2_	-	-	-	-	0.00000	-
Cu(pimp)_2_	-	-	-	-	0.00000	-
Zn(pimp)_2_	−99,731.257	−1.18563	−0.4894	−875,361.812	0.00000	−1.18563

**Table 4:** Data set of selected steric descriptors calculated from molecular docking data.

Complex	SlogP	HF	M_R_	Dipole Moment	V_surf_
H-pimp	3.1428	28.3960	10.7179	3.4950	0.00000
Ni(pimp)_2_	5.5234	-----------	7.9865	0.0000	0.00000
Co(pimp)_2_	4.5117	------------	9.4579	0.0000	0.00000
Cu(pimp)_2_	6.1117	-----------	6.1803	0.0000	0.00000
Zn(pimp)_2_	2.3745	96.1966	11.7197	16.9428	0.00000

## Data Availability

All data generated or analyzed during this study are included in this published article.
